# Dental Implications of Very Long‐Chain Acyl‐CoA Dehydrogenase Deficiency: A Comprehensive Case Report and Literature Review

**DOI:** 10.1002/ccr3.9670

**Published:** 2024-11-29

**Authors:** Abdullah Faraj Alshammari, Ebtsam Abdullah Aledaili, Jawaher Saad Alrimali, Bander Mushawwah Alhamazani, Khlood Abdulkader Alkurdi

**Affiliations:** ^1^ Department of Basic Dental and Medical Science, College of Dentistry University of Ha'il Ha'il Kingdom of Saudi Arabia; ^2^ Ministry of Health Ha'il Health Cluster Ha'il Kingdom of Saudi Arabia; ^3^ Dental Research Centre, College of Dentistry University of Ha'il Ha'il Kingdom of Saudi Arabia; ^4^ Ministry of Health, Qassim Health Cluster King Saud Hospital Unayzah Kingdom of Saudi Arabia; ^5^ Institute of Dentistry Queen Mary University of London London UK

**Keywords:** creatine kinase, hypoglycaemia, lipid metabolism, oral health, pediatric dentistry, very long‐chain acyl‐CoA dehydrogenase deficiency

## Abstract

This case report discusses the pathophysiology, clinical manifestations, and dental implications of very long‐chain acyl‐CoA dehydrogenase deficiency (VLCADD). If undiagnosed, VLCADD can be life‐threatening. Dental professionals must ensure patient safety through adequate knowledge, proper nutrition and glucose management, as well as genetic counseling in cases of consanguineous marriages.

## 1 Introduction and Literature Review

An estimated 9%–12% of medically compromised patients seek dental treatment annually, presenting distinct challenges to the dental profession [1]. The wide diversity and associated medical complications associated with such cases frequently increase the complexity of regular dental procedures. Dental healthcare workers (DHCWs), ranging from experienced professionals to those with limited experience, often express increased levels of anxiety when confronted with such cases. The complex nature of these medical conditions could increase the risk of complications and adverse events occurring during dental procedures [2].

The level of caution and concern experienced by DHCWs is further intensified when a patient's underlying condition is classified as a rare disease [1]. Very long‐chain acyl‐CoA dehydrogenase deficiency (VALCDD) is a rare medical condition. Due to its rarity, there is limited information available regarding the dental considerations associated with VALCDD, which consequently increases the anxiety experienced by DHCWs when individuals with VLCADD seek dental care.

This paper discusses a pediatric case of VALCDD who visited a dental department. It rationalizes the need to take specific measures while performing dental treatments and to ensure that DHCWs are familiar with appropriate measures to ensure the stability of such patients.

## 2 Search and Screening Strategies

An electronic search was systematically performed using online scientific servers (Google Scholar and PubMed) to retrieve available case reports published during the last 11 years (2013–2024). An additional manual search was performed by checking reference list for relevant studies. The keywords and search terms used were (Acyl‐CoA Dehydrogenase “Mesh” OR Very Long‐Chain OR Deficiency of Acadvl “Mesh” OR Vlcad‐H “Mesh” OR Very Long‐Chain Acyl Coenzyme “Mesh” OR Dehydrogenase Deficiency “Mesh” OR Very Long‐Chain Acyl‐Coenzyme Dehydrogenase Deficiency “Mesh” OR Vlcad‐C “Mesh” OR Very long‐chain acyl‐CoA dehydrogenase deficiency “Mesh” OR Acyl‐Coa Dehydrogenase “Mesh” OR Very Long Chain Deficiency) AND (Case report OR case series OR clinical details OR manifestation OR treatment).

**FIGURE 1:**
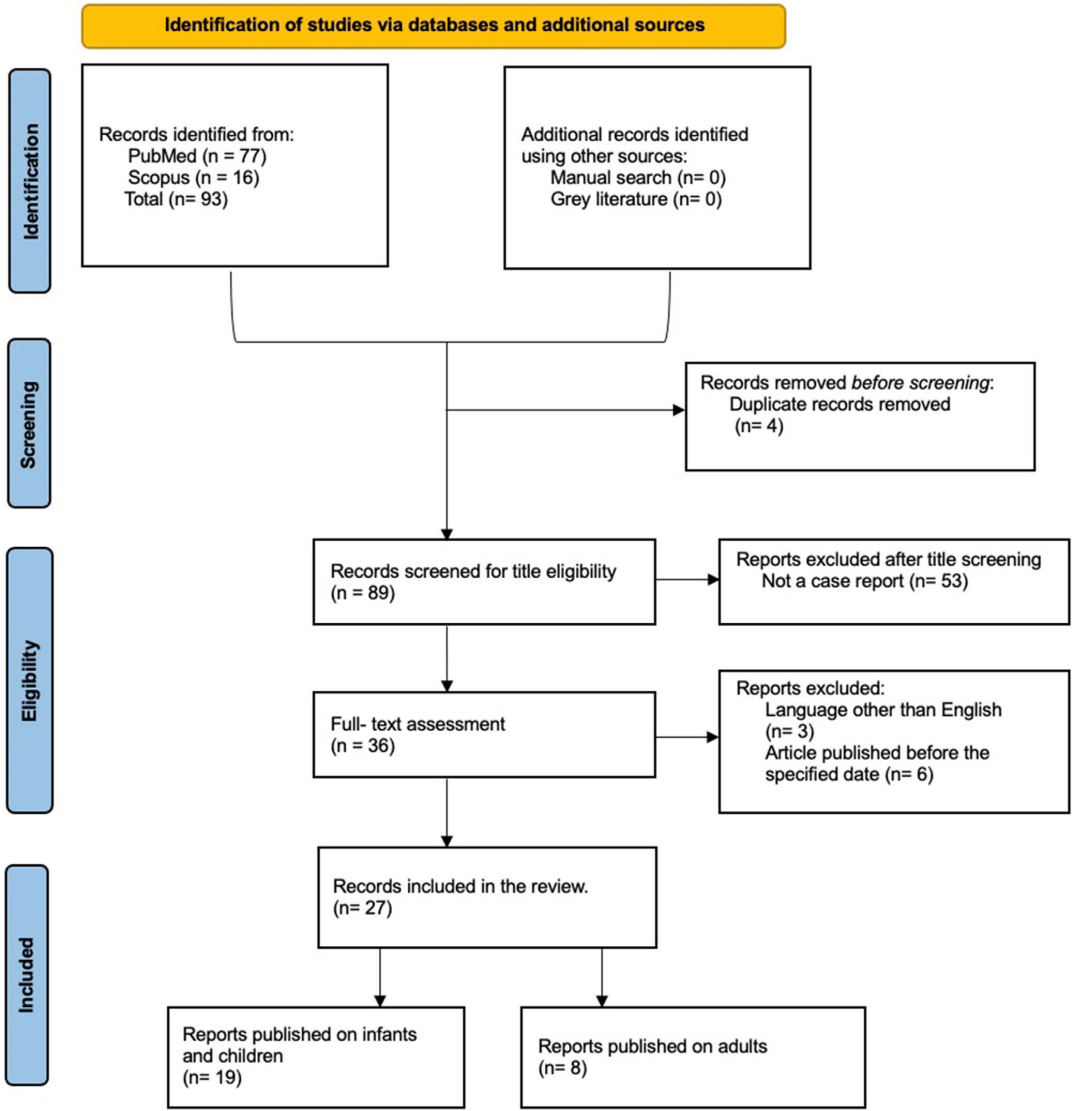
PRISMA 2020 flowchart illustrating the literature search and screening stages.

Studies were included based on a previously established eligibility criterion. Case reports or case series on patients with VLCADD were included, regardless of patient age and gender. Publications had to be written in English and published during the last 11 years. Publications on conditions other than VLCADD, duplicates, systematic reviews, dissertations, letters, personal opinions, book chapters, conference abstracts, and pilot studies were excluded. Figure 1 presents the screening process and reasons for study exclusions. The titles and abstracts of all retrieved papers were assessed independently by two researchers (KA and AA). When there was a dispute, a discussion was had to reach a decision. A third author (EA) was helped reach a consensus when required. The same researchers (KA and AA) independently evaluated the full‐text papers for inclusion. Consensus on eligibility was reached through discussion, and if necessary, a third researcher (EA) was engaged.

In total, 77 papers were retrieved from PubMed and 16 from Scopus. No additional papers were identified through the manual search or gray literature search. Of the 93 publications, four were excluded as they were duplicates. Two screening stages (titles/abstracts and text) were performed to evaluate the texts against the eligibility criteria and to retrieve the relevant data. Following the title and abstract screening, 53 papers were excluded for not being case reports or case series. Following the full‐text screening, a further five publications were excluded: one English, and four were published before the specified date range. The final number of included studies was 27. Nineteen infant cases, and eight involved adult cases.

The characteristics of the 28 included studies are presented in Table 1. The extracted domains revealed a mix of international publications, reflecting the wide geographical distribution of VALCDD. The data indicated that VALCDD is not associated with a specific age group or gender, with 51.6% (16/31) of retrieved cases occurring in males and 48.4% (15/31) occurring in females. Although VALCCD is considered a rare entity, the extracted data revealed an overall good prognosis, with only two cases (6.452%) reported to lead to fatality.

**TABLE 1:** Characteristics of the 27retrieved papers reflecting the wide geographical of VALCDD.

Author/Year	Country	Age	Gender	Consanguinity	Prognosis
Al‐Buaidi et al. (2023) [3]	Oman	14 Y	Male	NM	Good
Labella et al. (2023) [4]	Italy	17 Y	Male	No	Good
Singh et al. (2023) [5]	USA	1 D	Female	NW	Deceased
Peixoto et al. (2023) [6]	Portugal	10 Y	Male	NM	Good
Ahmed et al. (2022) [7]	Ireland	20 Y	Female	NM	Good
Alaei et al. (2022) [8]	Iran	4 Y	Female	NM	Good
Arunath et al. (2021) [9]	Sri Lanka	4 M	Female	Yes	Deceased
Bo et al. (2021) [10]	Japan	1.5 Y	Male	NM	Good
Fuseya et al. (2020) [11]	Japan	25 Y	Male	NW	Good
Kiss et al. (2020) [12]	Australia	38 W	Male	NM	Good
Wijayabandara et al. (2020) [13]	Sri Lanka	36 Y	Male	NM	Good
Yamada et al. (2019) [14]	Japan	31 Y	Female	No	Good
Hess et al. (2018) [15]	USA	3 Y	Male	NM	Good
Watanabe et al. (2018) [16]	Japan	6 Y	Male	NM	Good
Sebok et al. (2017) [17]	Hungary	46 Y	Female	NM	Good
Katz et al. (2016) [18]	Israel	2 D	Male	No	Deceased
Hisahara et al. (2015) [19]	Japan	18 Y	Male	NM	Good
Schwoerer et al. (2015) [20]	USA	6 W	Male	NM	Good
Stepien et al. (2015) [21]	UK	19 Y	Female	NW	Good
Yamamoto et al. (2015) [22]	Japan	12 Y	Female	No	Good
Murata et al. (2014) [23]	Japan	34 Y	Female	NM	Good
Takahashi et al. (2014) [24]	Japan	3 M	Male	NM	Deceased
Topcu et al. (2014) [25]	Turkey	13 Y	Female	NW	Good
Oliveira et al. (2013) [26]	Portugal	13 Y	Female	NM	Good
Sharef et al. (2013) [27]	Oman	3 D	Male	Yes	Good
Sedgick (2012) [28]	USA	2 D	Male	NM	Good
Roe et al. (2000) [29]	USA	3 Y	Female	No	Deceased

Abbreviations: D = day, M = month, NM = not mentioned, W = week, Y = year.

### 2.1 Very Long‐Chain Acyl‐CoA Dehydrogenase Deficiency

VLCADD is a fatty acid oxidation disease. Fatty oxidation diseases comprise a group of metabolic disorders. VLCADD is a rare genetic condition characterized by impaired fatty acid metabolism. It is considered life‐threatening and follows an autosomal recessive inheritance pattern [30]. According to a 2021 international screening campaign, VLCADD is the second most prevalent neonatal fatty acid metabolism defect, primarily affecting infants born to consanguineous parents. Its prevalence ranges from 1 in 30,000 to 1 in 400,000 newborns. Its prevalence exhibits a declining trend as individuals advance in age, becoming infrequent from around 6 years old. VLCADD lacks a distinct geographical distribution [31].

### 2.2 Pathogenesis

The etiology of VLCADD can be attributed to pathogenic mutations in the ACADVL gene, which encodes an essential enzyme referred to as very long‐chain acyl‐coenzyme‐A dehydrogenase. This enzyme is essential for the fatty acid degradation that occurs within the mitochondria [32]. Mitochondrial β‐associated fatty acid oxidation plays a crucial role in generating energy for cardiac and skeletal muscles, particularly during physiological stress [33]. Deficiencies in this gene cause a defect in the pathway of energy generation and impair fatty acid metabolism.

### 2.3 Clinical Picture

The National Organization for Rare Disorders has identified two stages of VLCADD. The first stage is early onset. Early onset is associated with a severe phenotype that, if not recognized and diagnosed, may result in a potentially life‐threatening myocardial weakness (cardiomyopathy). The second stage is later onset, associated with a less severe phenotype distinguished by recurrent episodes of hypoglycaemia. Individuals may exhibit symptoms associated with both onsets [31].

Symptoms associated with early onset VLCADD manifest shortly after birth, typically within days or weeks; however, some individuals may exhibit initial symptoms throughout early adolescence [34]. Infants with VLCADD exhibit symptoms indicative of hypoglycaemia, lethargy, and elevated blood ammonia levels [5]. At a later stage, individuals may exhibit muscular symptoms characterized by intermittent episodes of discomfort, exhaustion, and rhabdomyolysis [35].

Patients with VLCADD can exhibit signs of fatty acid infiltration and hepatomegaly [36]. Infants with VLCADD are also susceptible to cardiomyopathy, irregular cardiac rhythms, cardiorespiratory failure, and hypertrophy or dilation of the cardiac muscle (commonly referred to as hypertrophic or dilated cardiomyopathy). Cardiomyopathy can result in reduced myocardial contraction strength and diminished blood circulation efficacy in the pulmonary system and throughout the body, which, in turn, can manifest as heart failure. Specific symptoms experienced by patients vary based on the characteristics and severity of the condition and patient age [36].

### 2.4 Diagnosis

Following the introduction of an expanded newborn screening program, referred to as tandem mass Spectrometry technology (tandem MS), most infants with VLCADD are now diagnosed during the neonatal period, using blood and urine samples [37].

### 2.5 Treatment

The management of patients with VALCDD is tailored to their specific clinical profile and level of enzyme activity [38]. The recommended therapeutic interventions for individuals with VLCADD include dietary modifications and avoidance of prolonged fasting. The recommended diet is characterized by high carbohydrate content, increased medium‐chain triglyceride (MCT) intake, and decreased long‐chain triglyceride intake. Taking L‐carnitine supplements is potentially beneficial; however, further research is required to validate its effectiveness [16].

## 3 Case History/Examinations

A 4‐year‐old Saudi Arabian female was referred by her general dental practitioner to the Department of Pediatric Dentistry of a stomatology hospital in Saudi Arabia. The initial consultation was conducted in 2022, during which the patient, accompanied by her parents, exhibited symptoms of multiple dental caries. The patient described experiencing a dull pain triggered by eating that started 10 days ago.

The patient's medical history indicated a previous VLCADD diagnosis. The diagnosis by the neonatologists in corporation with geneticists was established at birth through tandem MS and molecular testing. The diagnostic tests identified a homozygous pathogenic variant with normative growth parameters. The patient was additionally diagnosed with concomitant chronic gastritis, and the medical notes revealed frequent episodes of lethargy, fatigue, gastroesophageal reflux, and vomiting. A clear chest with no pulmonary abnormalities was also noted. The abdomen was soft, non‐tender, and lax, with no evidence of organomegaly. The pediatric cardiological evaluation indicated tachycardia with overall normal echocardiography for the patient's age. The patient was conscious with no focal neurological deficits or psychological signs and was recommended to undergo annual follow‐ups.

The patient followed a rigorously controlled mixed diet with fat restrictions and a daily fluid intake of 1.2–1.6 L. The patient did not report any allergies. No familial incidence of VLCADD was disclosed; however, the patient's parents were first cousins. At the time of the dental consultation, the patient was on a specialized dietary regimen outlined by the nutritionist, entailing a daily caloric intake of 1440 kcal and consuming nine 200 mL scoops of a custom formula five times a day. The formula was composed of Nutricia Monogen formula, four scoops of 25 g CARBOCH, and two scoops of protein. The patient also consumed 20 mL of MCT oil once a day. The patient took a daily pharmaceutical prescription by the pediatrician, comprising an intravenous dose of Omeprazole (10 mg PO), with caproic acid (5 mL), and an IV infusion of 5% dextrose‐saline solution at a rate of 67 mL/h (measured based on weight). The patient's mother adhered to the prescribed dietary recommendations and took charge of administering the pharmaceuticals.

### 3.1 Investigations

**FIGURE 2:**
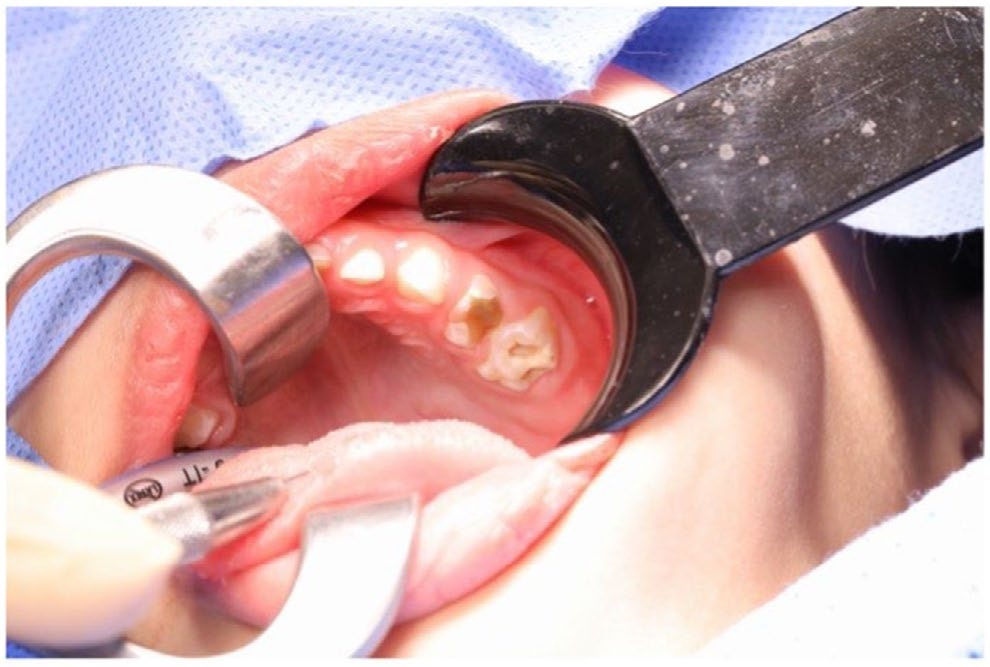
Clinical presentation before the intervention, illustrating the upper left quadrant of the patient's oral cavity and deep carious lesions on teeth #64 and #65.

**FIGURE 3:**
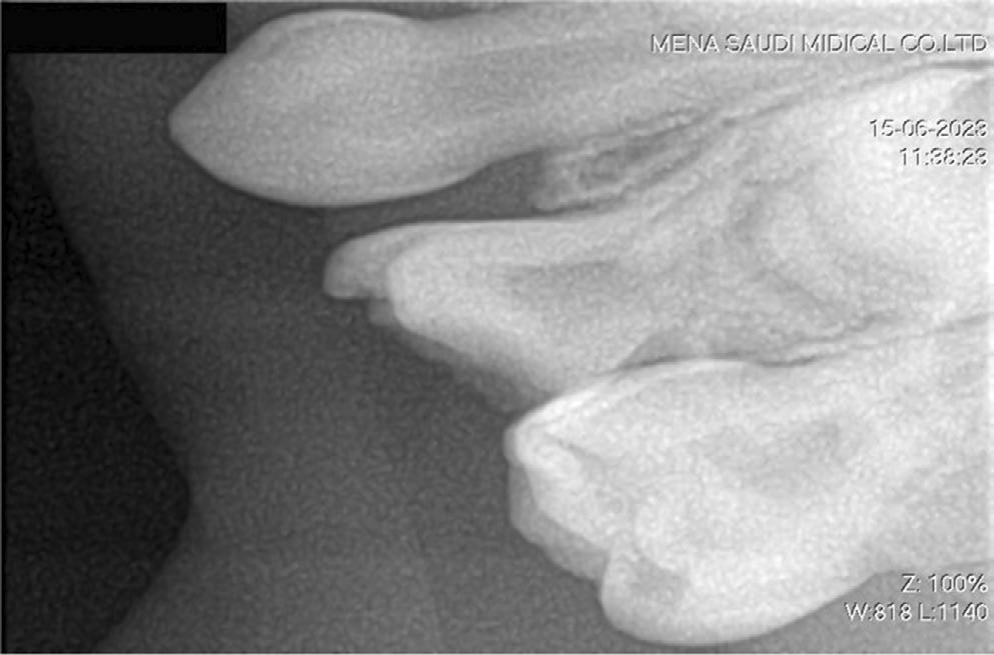
A radiological periapical X‐ray of the patient pre‐intervention illustrating the upper left quadrant of the oral cavity.

Despite the patient's uncooperative attitude, a comprehensive extraoral evaluation was performed by the pediatric dentist which revealed no substantial pathology, normal temporomandibular joint opening, and no swelling of the salivary gland or lymph node. However, perioral lip cracking and desquamation were observed on the right lower lip. The intraoral assessment indicated compromised oral hygiene and potential transient lingual papillitis. The mucosa appeared normal in color and texture, and salivary flow was normal. Upon dental examination, multiple carious lesions were identified in teeth #54, #55, #61, #64, #65, #74, #75, #84, and #85. Figure 2 presents the clinical picture during the dental examination, and Figure 3 presents the radiological periapical X‐ray. The standards for treating medically compromised patients state that treatment should be done under general anesthesia (GA). This standard was followed. Informed high‐risk consent was obtained from the parents.

Vital signs were recorded. The patient's weight and height indicated standard developmental rates (12 kg and 95 cm). Pre‐operative hematological and biochemistry tests were conducted, as presented in Table 2. The results revealed critically high levels of creatinine kinase (2678 U/L; normal range (NR) < 145 U/L) and markedly elevated levels of alkaline phosphatase (142 U/L; NR < 1 04 U/L), alanine transaminase (177 u/L; NR < 33 U/L) and aspartate transaminase (416 U/L; NR < 32 U/L).

**TABLE 2:** Pre‐intervention blood and histochemistry test results indicating critically high creatinine kinase and alkaline phosphatase levels and elevated alanine transaminase and aspartate transaminase levels.

Laboratory test	Value	Reference range
Biochemistry		
Creatine kinase (U/L)	2678	< 145
Aspartate aminotransferase (U/L)	345	< 34
Alanine aminotransferase (U/L)	311	< 49
Creatinine (umol/L)	32	49–90
Potassium (mmol/L)	4.60	3.5–5.1
Sodium (mmol/L)	143	136–145
Calcium (mmol/L)	2.41	2.08–2.65
Magnesium (mmol/L)	0.82	0.66–1.07
Prothrombin time	14.10	< 6
International normalized ratio	1.05	0.8–1
Partial thromboplastin time	38	26–40
Glucose random (mmol/L)	4.82	3.88–6.38
Hematology		
Total white cell count (cells/μL)	7290	4000–11,000
Hemoglobin level (g/dL)	12.10	10.9–15
Neutrophil count (cells/μL)	85000	36000–73000
Lymphocyte count (cells/μL)	11700	18000–48000

### 3.2 Treatment

**FIGURE 4:**
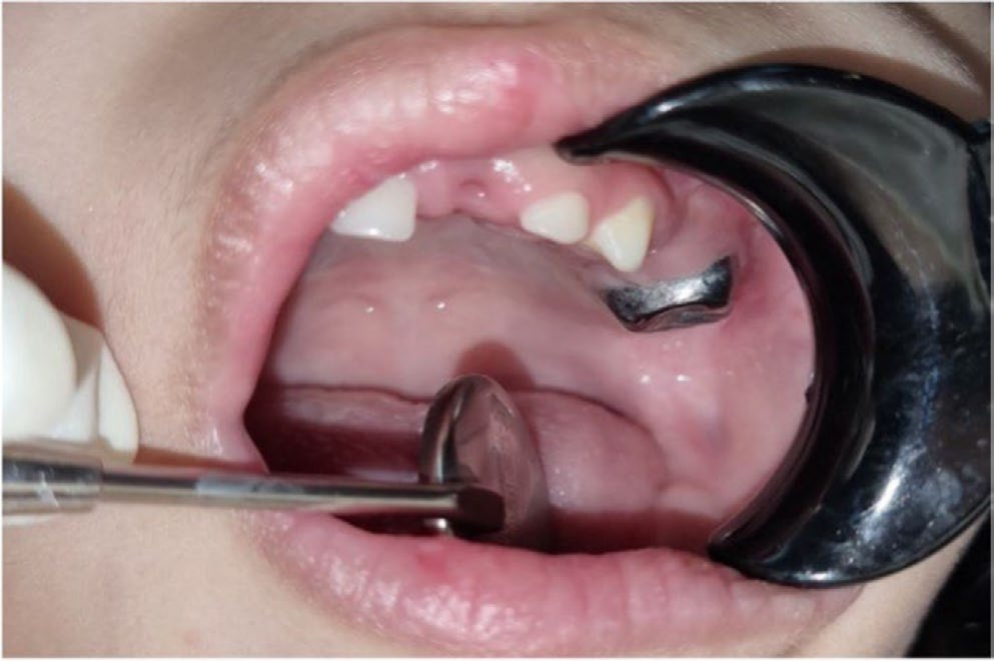
Clinical presentation post‐intervention, illustrating the upper left quadrant of the patient's oral cavity. Extractions were performed for teeth #61 and #64, and a stainless‐steel crown was prepared for #65.

The patient was assigned for dental intervention under GA, during which preventive and reparative procedures were executed. The patient was admitted by the pediatric dentist 1 day before the procedure and placed on a ‘nothing by mouth’ scheme. The patient was given an IV infusion of 5% dextrose‐saline solution at 44 mL/h and amoxicillin syrup (3.5 mL OP BID). Intraoperative glucose levels were monitored throughout the procedure using the random blood sugar test. A reduction was noted from 200 to 140 mg/dL, which was rectified. Several procedures were performed: preventative resin restoration for teeth #54 and #85, stainless steel crown for teeth # 55 and #65 (presented in Figure 4), pulpotomy followed by SCC for teeth #74, #75, and #84, and extractions on teeth #61 and #64. Post‐operative care was initiated in a specialized recovery unit. Due to the patient's age and non‐compliant attitude, she was transferred to the pediatric surgical ward for augmented monitoring and dietary management.

The patient was discharged on the same night of the operation, as her parents signed a consent for discharge against medical advice. However, the patient was readmitted within 24 h due to complications that included recurrent vomiting, dehydration, and no tolerance for oral feeding.

### 3.3 Outcome and Follow‐Up

The complications were managed with fluids, and the patient's oral intake improved after 8 h. The patient was discharged after 3 days once hematological tests confirmed her creatine kinase levels were average, and the symptoms had subsided. The patient was given a detailed therapeutic guideline that included a prescription for domperidone (5 mg OP TID), and her parents were given guidance on how to improve her quality of life, including increasing her fluid intake and following guidelines for oral hygiene and fluoride varnish application. A follow‐up consultation 2 weeks post‐discharge confirmed the patient's stabilized condition, enabling ongoing clinical monitoring. There were no adverse events or side effects associated with the drug prescribed.

## 4 Discussion

The prevalence of rare diseases can expose DHCWs to relatively unexplored fields. This, in turn, can cause anxiety and work‐related stress. That is because, rare diseases are challenging to diagnose and treat accurately. The rarity of these diseases means they are less likely to be covered extensively in medical education, leading to uncertainty and anxiety when faced with such cases. Therefore, there is a continuous need for practitioners to gain expertise in these rare diseases. Opportunities to gain this expertise emerge from documenting these rare diseases through case reports and case series. Additionally, conference discussions and verbal knowledge exchanges can serve to raise awareness among healthcare workers. These opportunities will help sustain DHCWs' confidence in tackling rare cases. The establishment of the International Rare Diseases Research Consortium in 2011 has facilitated the development of novel therapies and diagnostic approaches. However, dental considerations tend to be overlooked.

Although there is no established evidence indicating that VLCADD directly leads to specific dental conditions, one study reported the death of a young female patient with VLCADD while undergoing routine dental treatment [29]. This occurred due to the patient undergoing perioperative oral fasting without intravenous administration of glucose. Without glucose administration, the patient's body was unable to compensate for the lack of energy from fat breakdown, resulting in a potentially fatal energy crisis. When providing dental care, it is essential that DHCWs are aware of a patient's distinct medical requirements, as individuals with VLCADD are prone to hypoglycaemia, which can be induced by fasting or stress [39]. This published case was read by all the DHCWs who treated the current patient, and they were aware of the considerations and complications related to children with metabolic and endocrine disorders, namely that such individuals cannot endure extended intervals without nutrition. This knowledge helped facilitate a successful patient outcome.

The systematic search revealed that VLACDD has been reported in multiple geographic locations. This indicates it is non‐specific regarding ethnicity. The published literature indicates that cases originating from Europe, America, and Australia typically exhibit milder VLACDD characteristics compared to cases originating from Asia, where our patient originated [39]. A potential reason for this observation could be genetic variability or differences in environmental, nutritional, or healthcare factors across regions. Additionally, the literature demonstrated that the clinical manifestations of VLCADD differ based on the age of onset (early and late). Although the patient discussed in this report was young (4 years old), she experienced symptoms associated with both onsets. The patient experienced tachycardia and myopathy; however, no liver dysfunction was noted. Mixed symptoms have been reported previously. In 2018, Obaid et al. reported a patient who exhibited symptoms of both onsets [40].

A study published by Merinero et al. in 2017 suggested that starting dietary changes early in cases of VLCADD while waiting for enzymatic and molecular assessment results can enhance patient outcomes [39]. However, in 2018, Obaid et al. found that implementing dietary changes shortly after being diagnosed with severe symptoms did not affect patient outcomes [40]. For our patient, the treatment plan was tailored to her needs as soon as a definitive diagnosis was reached.

Our patient had a parental history of close relativity. Consanguineous marriages significantly increase the inherent risk of genetic illnesses [9]. This is because there is a higher chance that both partners have the same recessive gene mutations, which significantly increases the chances of any children being affected by uncommon genetic disorders. This emphasizes the importance of making well‐informed choices regarding marriage and family planning, as well as the essential role of gene counseling.

In the holistic and comprehensive management of our patient with VLCADD, the collaboration of various healthcare professionals was essential. Neonatologists and geneticists played a pivotal role in diagnosing VLCADD early, which allowed for timely intervention and the prevention of life‐threatening complications. Pediatricians were involved in overseeing the patient's overall health, coordinating care between specialties, and monitoring growth and development. The nutritionist ensured that the patient maintained appropriate glucose levels and followed a diet tailored to the specific metabolic needs associated with VLCADD. The pediatric dentist took care of the patient's oral health, addressing dental concerns while considering the broader metabolic condition and ensuring treatments aligned with nutritional management. This multidisciplinary approach was critical in providing comprehensive care and ensuring the patient's overall well‐being.

## 5 Conclusion

In light of these findings, the significance of knowledge exchange within dentistry cannot be underestimated. By exchanging experiences related to rare diseases, professionals can cultivate a more extensive reservoir of information, which improves the quality of care provided to patients.

### 5.1 Limitations

One limitation of this report is the lack of photographic and radiologic illustrations of other quadrants of the oral cavity, as only the left upper quadrant is shown.

## Author Contributions

**Abdullah Faraj Alshammari:** conceptualization, resources, supervision, writing – original draft. **Ebtsam Abdullah Aledaili:** conceptualization, investigation, methodology, supervision. **Jawaher Saad Alrimali:** formal analysis, investigation, methodology, visualization. **Bander Mushawwah Alhamazani:** methodology, resources, software, visualization. **Khlood Abdulkader Alkurdi:** formal analysis, software, validation, writing – review and editing.

## Ethics Statement

The study protocol for this case report was reviewed and approved by the Ethics Research Committee of the University of Hail, in compliance with institutional and national ethical standards for research involving human subjects.

## Consent

Written informed consent was obtained from the patient for the publication of this case report, including all clinical information and accompanying images. The patient was made aware of the content and has approved the final version of the manuscript.

## Conflicts of Interest

The authors declare no conflicts of interest.

## Data Availability

All relevant data supporting the findings of this case report are included within the manuscript. No additional data are available beyond what is presented, which supports the conclusions drawn in the report.
